# Do medical outpatients want 'out of hours' clinics?

**DOI:** 10.1186/1472-6963-5-47

**Published:** 2005-06-24

**Authors:** Claire L Feeney, Nicola J Roberts, Martyn R Partridge

**Affiliations:** 1 Department of Respiratory Medicine, NHLI at Charing Cross Hospital, Imperial College, St Dunstans Road, London W6 8RP, UK

## Abstract

**Background:**

Patient choice is a major theme in current healthcare delivery. Little is known about patients' wishes regarding the timing of medical outpatient clinics.

**Methods:**

A questionnaire survey of 300 sequential patients attending cardiac and respiratory clinics to determine patients preferences for out of hours and weekend outpatient clinics. (Out of hours defined as a clinic after 5 pm on Mon – Fri)

**Results:**

Two hundred and 64 patients completed the survey of which 165 (62.5%) wanted either an out of hours clinics or a weekend clinic. Sixty four (38.8%) specifically stated that this was because of work commitments but for many others, the reasons given were easy to justify.

**Conclusion:**

Current provision for outpatient consultation may not be convenient for many patients with heart and lung disease. A fuller evaluation of the cost and benefits of more flexible clinic hours is now needed.

## Background

Patient choice is a key part of current U.K. National Health Service (NHS) Strategy [1]. At a time when the population has access to 24 hour banking and to supermarkets which are open 24 hours a day, specialist consultations in U.K. hospitals for non-emergency conditions remain largely confined to the hours of 9-5 pm. In this regard it would appear that the NHS has failed to keep pace with a society which generally demands fast, convenient and 24/7 services.

The Department of Health's 10 year plan has echoed these sentiments and specifically states that the NHS has been too slow to change its ways of working to meet modern expectations in this consumer age. While it recognises that the NHS is under funded, money alone can only be a starting point. Cost-effectiveness, it states, is the key to success and this can only be achieved if available resources are used to achieve real benefits for patients. Clearly, the redundancy of most outpatient clinics after 5 pm and during the weekend is not an efficient use of resources, nor is it a modern way of working.

Medical practice nowadays strives to be patient-centred [2]. There is good evidence that involving patients in decision-making and listening to the patient voice improves health outcomes and patient satisfaction. Hence, when we undertook an extensive literature review, we were surprised to find no published work concerning medical patients' views on out of hours clinics in secondary care. Given the current government proposals we felt it necessary to explore patients' views about out of hours clinics amongst patients attending medical outpatients' clinics in a central London teaching hospital.

After embarking on our study of medical outpatients we subsequently found two other reports on patients' opinions on out of hours ophthalmology clinics. The first is a study of patients with ophthalmological conditions attending a hospital in Leeds and Bristol which looked at whether patients found their current appointments and surgery times convenient or inconvenient [3]. They found that 89% of their patients were apparently happy with their current appointment times but when asked about alternatives would find Saturday mornings convenient. The second study was a critique of the first and reported the responses of 100 consecutive patients attending Saturday morning ophthalmology clinics at an outer London hospital [4]. They were asked whether they preferred a Saturday morning clinic, a regular 9-5 appointment, or whether they didn't mind either. They found that 50% had no preference, 41% preferred a Saturday morning and only 9% preferred a 9-5 pm weekday appointment. Both these studies have their limitations in that the first essentially asked about convenience not preference and the second may be affected by bias in that verbal responses alone were reported of a select group of patients i.e. only those already attending a Saturday morning clinic. These studies are based in ophthalmology clinics and may not be representative of all patients attending hospital outpatient clinics. Our study is based on respiratory and cardiology outpatient clinics. These two disease areas account for a major part of the burden of medical illness in the UK with 8 million people having lung disease alone.

## Methods

Centre of data collection: Charing Cross Hospital, London

Clinics: Respiratory, Monday morning, Wednesday morning and Thursday afternoons; Cardiology, Thursday morning

Duration of data collection: 6 months

Patient Profile: Outpatients routed through the central booking office (excluding rapid or open access respiratory and cardiology clinics).

300 sequential attendees at Cardiorespiratory clinics were asked to complete a questionnaire [see additional file 1] to determine their opinions regarding access to out of hours (defined as after 5.00 pm) and weekend outpatient clinics. They were then asked to give up to five reasons as to why they might be interested in such clinics. The patients included a mixture of patients attending for the first time and those attending for follow-up appointments. The reception staff were specifically asked to give the questionnaire to all patients who had not previously received them. The items selected for inclusion in the questionnaire had been raised repetitively in another study (not yet published) looking at reasons why patients did not attend clinics (forgetfulness, long waits, seeing a different doctor each time, nowhere to park, time off work, no one available for childcare).

Data was analysed using the statistical package SPSS (version 12.0), however as this was an observational study power calculations were not calculated and data was only subjected to descriptive analysis

## Results

264 completed questionnaires were obtained (response rate 88%) from 124 males and 122 females with a mean age (SD) of 58 years (16.94). 144 of the patients were attending with a respiratory condition (amongst which were 52 with asthma, 18 with sleep related breathing disorders, 13 with unexplained cough) 65 with a cardiac condition (25 with ischaemic heart disease, 12 with arrhythmias, 9 with hypertension), and the remainder did not state their condition or had an ill-defined medical problem.

165 patients (62.5%) wanted either an out of hours clinic or a clinic on a Saturday or Sunday. Of the reasons given, 81 patients (49.1%) said it was the greater flexibility that such clinics offered with 64 (38.8%) specifically stating that it was because they worked during the day. Forty one (24.8%) responded that it was easier for them to get to the hospital in the evening because someone could drive them there. The same number responded that it was easier to get someone to accompany them in the evening, and 22 (13.3%) stated that the reason was because they had family commitments during the day. 18 patients listed other reasons (see Table 1) which included important practical issues such that parking restrictions in the environs of the hospital would not apply in the evening and at weekends.

**Table 1:** 'Other' Reasons volunteered by patients for wanting out of hours clinics

Reason	Number of patients
I am a shiftworker or self-employed so out of hours clinics would be more convenient	4
Less traffic and less stressful to get to the hospital	3
Parking restrictions would not apply	3
I would not have to finance child care at these times	3
Waiting times would probably be reduced	2

When the responses were sub-analysed according to whether the respondent was over or under the age of 65 years, 152 patients were seen to be under the age of 65. Of those 152 patients, 110 (72.4%) expressed a preference for out of hours clinics, whereas amongst the 100 patients aged over 65, 49 (49%) expressed such a wish. Not surprisingly amongst those aged under 65, the commonest reason stated for why they were interested in an out of hours clinic was that they worked during the daytime, and this response was given by 58 out of the 110 of the patients (53%)

The responses also varied according to the medical condition of the patient. Of the 144 patients attending with a respiratory condition, 97 (67.4%) wanted out of hours clinics, compared to 36 out of the 65 (55.4%) patients attending with a cardiac condition. However, the mean age of respiratory patients was 55 years compared to 65 years in the cardiac group.

Of those patients who wanted out of hours clinics, weekend clinics appeared to be consistently more popular than evening clinics and this trend was upheld even when the responses were analysed according to age, gender and medical condition.

## Discussion

The majority of medical outpatients we questioned would like more flexible timing of clinics. The reasons expressed to justify such a preference appear to be entirely reasonable, such as work commitments during the day. An extension of hours for routine consultations may therefore enhance care for those who are in employment. It also represents a logical fuller use of outpatient consultation facilities. However, out of hours clinics involve more than a doctor-patient consultation. Additional reception and nursing staff are likely to be needed and if the consultation is to be truly effective for the patient they would need to be able to have simple investigations like blood tests, ECG and imaging tests at the same time. Further economic evaluation of the cost of making such services available would be necessary before the widespread introduction of out of hours clinics. However, not all outpatients require investigations and it may not be difficult to construct a list of conditions where patient attending for follow-up can be offered out of hours services without other departments needing to be available.

The patients surveyed in this study were patients with medical conditions attending an inner city London teaching hospital. In a patient centred health service similar studies should probably be done amongst those with other conditions living in other areas.

The response rate for our study was high and reasons for non response are not known but could include the patient having gone straight into see the doctor and having no waiting period in which to complete the form. In a multicultural society some may have had difficulty with a questionnaire in the English language and up to 15% of patients attending some UK out patient clinics may be functionally illiterate [5]. The response rate is unlikely to have altered the validity of the conclusions which are similar to one study in an ophthalmology clinic [4] but dissimilar to another [3]. The differences almost certainly reflect the question which is asked (stating actual preferences versus stating satisfaction with what you have) and how it is asked and by whom.

## Conclusion

We feel that this is an interesting and important area of research with the results suggesting that the hours of opening of traditional medical outpatients clinics should be extended. Each hospital and each speciality should probably survey their own clientele, but to enable comparisons to be made some form of standardised wording should be used and the questionnaire itself should be included in any subsequent report.

## Competing interests

MRP, CF and NJR do not have any significant competing interests (financial or non-financial).

## Authors' contributions

MRP developed the study design and protocol. NJR and CF were responsible for implementing the study and analysing the results. All contributed to drafting the report and approved final manuscript.

Additional file 1:

## Pre-publication history

The pre-publication history for this paper can be accessed here:



## Supplementary Material

Additional File 1 A copy of the questionnaire used for this study. Click here for file
