# Supplementary material for: Do medical outpatients want 'out of hours' clinics?
Source: BMC Health Serv Res. 2005 Jun 24;5:47. doi: 10.1186/1472-6963-5-47 (PMC1173106; doi:10.1186/1472-6963-5-47)
Supplement: Additional File 1 — A copy of the questionnaire used for this study. [file 1472-6963-5-47-S1.doc]

# PLEASE HELP US TO HELP YOU BY COMPLETING

# THIS SHORT QUESTIONNAIRE

#

The Health Service is continually trying to be more patient centred. There is frequent talk about clinics being held at more convenient times. However we have no idea how many people attending our clinics would want or need of out of hours clinics, even if it was possible for us to arrange them. We are keen to know how many people attending this clinic would be interested in having the option of an ‘out of hours’ clinic appointment (that is to say after 5 pm Monday to Friday) or a clinic at the weekends. We would appreciate it if you could take the time to answer the questions below while you are waiting and then place the completed form in the box when you have finished.

**The questionnaire is anonymous**.

1) Are you interested in the possibility of an ‘out of hours’ clinic (a clinic after 5pm on Mon - Fri)? Yes  No 

2) Are you interested in the possibility of a clinic on Saturday or Sunday?

Yes  No 

3) If you answered yes to either Question 1 or 2 or both please tell us why are you interested in an

‘out of hours’ clinic? **Please tick all that apply to you**

a) I work in the daytime 

b) I have family commitments during the day 

c) It is easier for me to get to the hospital in the evening because someone can drive me there 

d) It is easier to get someone to accompany me in the evening 

e) I would like the greater flexibility to my day that an ‘out of hours’ clinic offers 

f) Other reason. (*Please state) {Continue overleaf if necessary}* 

…………………………………………………………………………

…………………………………………………………………………

4) Please tell us what condition brings you to the clinic (using your own words if necessary)

for example, asthma, previous heart attack, scarring of the lungs, palpitations, COPD,

a persistent cough, tumour, recent pneumonia, TB , blood pressure etc

…………………………………………………………………………………………………....

5) Finally please tell us your gender ( circle **Male** / **Female**  ) and **age……**……………………..

THANK YOU!!

PLEASE LEAVE THE COMPLETED FORM IN THE BOX
